# Supplementary material for: The causal relationship between the human gut microbiota and pyogenic arthritis: a Mendelian randomization study
Source: Front Cell Infect Microbiol. 2024 Nov 26;14:1452480. doi: 10.3389/fcimb.2024.1452480 (PMC11629706; doi:10.3389/fcimb.2024.1452480)
Supplement: Supplementary file 1 [file DataSheet1.zip › mendelian test/ebi-a-GCST90027488.csv_leave_one_out.pdf]

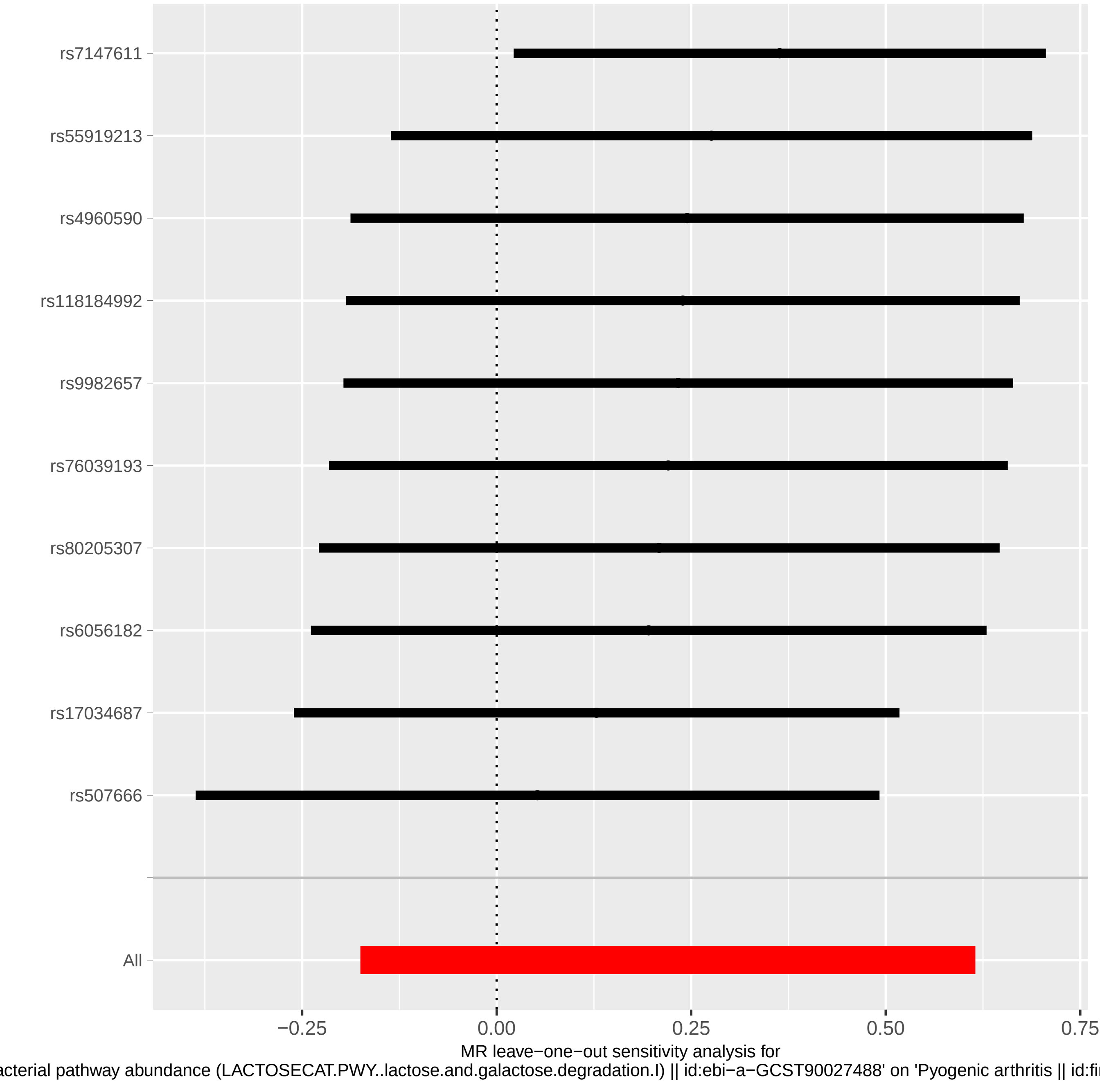

rs7147611
rs55919213
rs4960590
rs118184992
rs9982657
rs76039193
rs80205307
rs6056182
rs17034687
rs507666
All
−0.25
0.00
0.25
0.50
0.75
MR leave−one−out sensitivity analysis for
acterial pathway abundance (LACTOSECAT.PWY..lactose.and.galactose.degradation.I) || id:ebi−a−GCST90027488' on 'Pyogenic arthritis || id:fi
